# Novel essential amino acid supplements enriched with L-leucine facilitate increased protein and energy intakes in older women: a randomised controlled trial

**DOI:** 10.1186/s12937-017-0298-6

**Published:** 2017-11-28

**Authors:** Theocharis Ispoglou, Kevin Deighton, Roderick FGJ King, Helen White, Matthew Lees

**Affiliations:** 1 Carnegie School of Sport, Leeds Beckett University, Headingley Campus, Fairfax Hall, Leeds, LS6 3QS UK; 2 School of Applied and Clinical Sciences, Leeds Beckett University, CL413 Calverley Building Civic Quarter, Leeds, LS1 3HE UK; 3 Carnegie School of Sport, Leeds Beckett University, Headingley Campus, Fairfax Hall, Leeds, LS6 3QS UK

**Keywords:** L-leucine, Energy, Protein, Sarcopenia, Appetite, Hormones

## Abstract

**Background:**

Inadequate protein intake (PI), containing a sub-optimal source of essential amino acids (EAAs), and reduced appetite are contributing factors to age-related sarcopenia. The satiating effects of dietary protein per se may negatively affect energy intake (EI), thus there is a need to explore alternative strategies to facilitate PI without compromising appetite and subsequent EI.

**Methods:**

Older women completed two experiments (EXP1 and EXP2) where they consumed either a Bar (565 kJ), a Gel (477 kJ), both rich in EAAs (7.5 g, 40% L-leucine), or nothing (Control). In EXP1, participants (*n* = 10, 68 ± 5 years, mean ± SD) consumed Bar, Gel or Control with appetite sensations and appetite-related hormonal responses monitored for one hour, followed by consumption of an ad libitum *breakfast* (ALB). In EXP2, participants (*n* = 11, 69 ± 5 years) ingested Bar, Gel or Control alongside an ALB.

**Results:**

In EXP1, EI at ALB was not different (*P = 0.674*) between conditions (1179 ± 566, 1254 ± 511, 1206 ± 550 kJ for the Control, Bar, and Gel respectively). However, total EI was significantly higher in the Bar and Gel compared to the Control after accounting for the energy content of the supplements (*P < 0.0005*). Analysis revealed significantly higher appetite Area under the Curve (AUC) (*P < 0.007*), a tendency for higher acylated ghrelin AUC (*P = 0.087*), and significantly lower pancreatic polypeptide AUC (*P = 0.02*) in the Control compared with the Bar and Gel. In EXP2, EI at ALB was significantly higher (*P = 0.028*) in the Control (1282 ± 513 kJ) compared to the Bar (1026 ± 565 kJ) and Gel (1064 ± 495 kJ). However, total EI was significantly higher in the Bar and Gel after accounting for the energy content of the supplements (*P < 0.007*).

**Conclusions:**

Supplementation with either the Bar or Gel increased total energy intake whether consumed one hour before or during breakfast. This may represent an effective nutritional means for addressing protein and total energy deficiencies in older women.

**Trial registration:**

Clinical trial register: retrospectively registered, ISRCTN12977929 on.

## Background

Optimum protein and energy intakes in older adults are important for promoting good health [1–3], recovery from illness [4] and maintenance of function [5–7]. When requirements are not met the risk of sarcopenia increases [8, 9]. Characterised by a progressive decline in skeletal muscle mass and strength [10, 11], sarcopenia is known to increase morbidity, mortality, and associated health care costs [12]. Sarcopenia has been recently recognised as a disease [13], while its high prevalence has brought attention towards identifying strategies that can assist older individuals in meeting their dietary needs. It has been suggested that current recommendations of 0.8 g^.^kg^-1.^d^−1^ for dietary protein should be increased to 1.0–1.6 g^.^kg^-1.^d^−1^ and that a higher protein threshold of at least 25–30 g protein per-meal should be encouraged in this age group to promote anabolism [8, 14–19]. However, reductions in taste and appetite [20, 21], slower gastric emptying, and changes in appetite-regulating hormones [22] that can occur irrespective of disease [23] present unique challenges to this population. A diminished anabolic response to protein [15, 24, 25] and the increased requirements caused by inflammatory and catabolic responses to diseases of ageing may compound this further. It is worth noting that a large proportion older individuals do not even meet the existing recommendations for protein, however deficiencies tend to be larger in women than in men [26–28]. Taking into account the higher frailty rates that are observed in women with lower protein intakes [29, 30], it is imperative that adequate protein intake is maintained with advancing age. Paradoxically, protein also has a known and well-established satiating effect that can compromise dietary energy intake [17, 31, 32], which adds complexity to interventions that seek to increase both energy and protein intakes.

In response, a number of nutritional interventions involving supplementation with protein or essential amino acids (EAAs) have so far shown ambiguous results; some indicating promise [3, 7, 33–36] but others not [37, 38]. Supplementation with the essential amino acid L-leucine (leucine) has attracted specific interest due to its crucial role in the regulation of muscle protein synthesis [39, 40]. In particular, there is general agreement that older individuals have a higher leucine threshold and that they would benefit from larger amounts of leucine either within a meal or as a protein/EAAs supplement [7, 17, 36, 41–45]. However, there has been little consideration of the satiating effects of EAAs-based nutritional supplements enriched with leucine and no studies have examined the impact on appetite and concurrent mealtime energy intake nor the practical aspects of palatability. Therefore, the main aim of the current crossover design study was to investigate to what extent acute supplementation of EAAs-based nutritional supplements containing 7.5 g of EAAs (40% leucine) affects the appetite and subsequent dietary energy intake of older women. A secondary aim was to investigate the potential mechanism of action through the measurement of appetite-regulating gut hormones acylated ghrelin, pancreatic polypeptide (PP) and peptide tyrosine tyrosine (PYY).

## Methods

This investigation involved two experiments that were conducted in accordance with the guidelines laid down in the Declaration of Helsinki. All procedures were approved by the University Faculty Research Ethics Committee and written informed consent was obtained from all participants. The trial was registered at ISRCTN registry with a trial registry number of ISRCTN12977929. Study participants were independently living female older adults aged between 60 and 80 years, free from vascular and metabolic disease, and of good health. Participants were excluded if they smoked, had used estrogens within the previous three months, or were lactose intolerant. In each study, participants were recreationally active but were not engaged in a regular programme of exercise training.

For both experiments, participants were asked to record all food and fluid consumed in the 24 h prior to the first experimental trial and replicate this for all subsequent trials. Participants were also asked to avoid alcohol and intensive physical activity during the same time period. All trials commenced between 07:30 am and 09:00 am after an overnight fast of at least 10 h. Participants exerted themselves minimally when travelling to the laboratory, using motorised transport where possible. Verbal confirmation of the dietary and exercise standardisation was obtained at the beginning of each experimental trial.

### Preliminary screening and anthropometry

In both experiments, the first visit to the laboratory involved an initial briefing and screening process. Participants were provided with information on the study procedures and given instructions with regards to the dietary replication and pre-test requirements. Baseline stature (to the nearest cm) and body mass (to the nearest kg) were recorded by a stadiometer (SECA Alpha, Birmingham, UK) and scales (SECA Alpha 770, Birmingham, UK). Resting heart rate (RHR), systolic and diastolic blood pressure (SBP and DBP, respectively) were measured alongside these variables, using a heart rate monitor (Polar, Kempele, Finland) and a sphygmomanometer (Accoson, Essex, UK). Self-reported physical activity levels were estimated using the short-form International Physical Activity Questionnaire (IPAQ) [46]. The weekly total estimated energy expenditure for our sample group was 5437 ± 6286 MET minutes with a range of 537–17,940 MET minutes. The descriptive characteristics for both study populations are provided in Table 1.

**Table 1:** Descriptive characteristics for the study populations in experiment one and two. Data presented as mean ± SD

Variable	Experiment one (*n* = 10)	Experiment two (*n* = 11)
Age (years)	68.4 ± 4.5	69.3 ± 5.2
Height (cm)	161.8 ± 5.4	161.7 ± 5.2
Body mass (kg)	65.2 ± 8.1	65.0 ± 7.7
BMI (kg^.^m^2^)	24.9 ± 3.1	24.9 ± 3.0

### Experimental protocol

In the first experiment (EXP1), older women (*n = 10*) completed three trials each separated by a minimum of one week in a randomised, crossover design. Appetite sensations and plasma acylated ghrelin, PP and PYY responses to a 37.5 g bar (BAR, 565 kJ), a 50 ml gel (GEL, 478 kJ) or nothing (CON) were investigated over the course of one hour, followed by an ad libitum breakfast (ALB). Both the BAR and GEL provided 7.5 g of EAAs with the same amino acid concentration as the 40% leucine EAAs mixture previously described by Ispoglou et al. [7]. The EAAs were purchased from Fagron UK Ltd., and the remaining materials from a main supermarket chain in UK (J Sainsbury plc). The finished products were developed in collaboration between our institute and a Product Developer to the Food Industry based at Askham Bryan College (UK). The key nutritional information per 100 g of finished product was:

- For the BAR: energy 1511 kJ, fat 8.2 g, carbohydrate 47.5 g, protein 25.4 g of which 15 g was EAAs, fibre 2.8 g, salt 0.2 g.

- For the GEL: energy 967 kJ, fat 0.0 g, carbohydrate 44.7 g, protein 15 g which was entirely due to the EAAs content, fibre 0.5 g, salt 0.2 g.

Baseline appetite perceptions and a baseline blood sample were collected five minutes prior to each condition, with participants instructed to consume each supplement within a five minute period. Once the breakfast was consumed, participants were asked to provide final appetite and palatability perceptions.

In the second experiment (EXP2), older women (*n = 11*) completed three trials separated by a minimum of one week in a randomised, crossover design. Participants were asked to consume either a BAR, GEL, or CON immediately prior to an ALB meal. Appetite and palatability ratings were obtained five min prior to each condition, with participants asked to provide postprandial ratings 20 min after starting the breakfast.

**Fig. 1:**
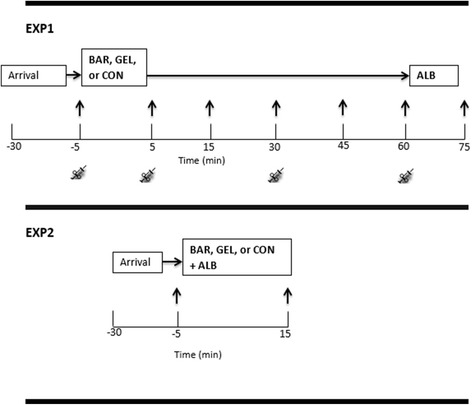
A schematic representation of the design for experiment one (EXP1) and two (EXP2). The three conditions consisted of either a bar (BAR), gel (GEL), or nothing (CON). Black arrows = appetite rating assessment; syringe picture = blood samples. Ad libitum *breakfast* (ALB)

The allocation sequence was computer-generated using a freely available online tool (GraphPad). Recruitment stopped when the required sample size of participants was achieved. An overview of the experimental design and testing procedures for both EXP1 and EXP2 is provided in Fig. 1.

### Ad libitum breakfast

In both experiments, the ad libitum breakfast was identical, with an energy density of 4.9 kJ/g and a macronutrient composition of 59% carbohydrate, 18% protein and 23% fat. Meal preparation involved mixing of 54 g of porridge oats (Oatso Simple Original, Quaker Oats) with 292 ml semi-skimmed milk. The mixture was then cooked in a microwave for two and a half minutes at 700 W. Participants consumed the breakfast in isolation to avoid any social influence on food intake. A bowl of the aforementioned meal was provided by an investigator and participants were instructed to eat until ‘comfortably full’, with no time limit set for eating. This bowl was replaced before the participant had emptied it, with minimal interaction and this process continued until the participant was comfortably full. Food intake was calculated as the weighted difference in food before and after eating [47].

### Appetite and palatability assessment

In both studies, appetite perceptions (hunger, satisfaction, fullness and prospective food consumption) were measured using 100 mm visual analogue scales with descriptors anchored at each end [48]. A composite appetite score was calculated as the mean value of the four appetite perceptions after inverting the values for satisfaction and fullness. Palatability ratings (visual appeal, smell, taste, aftertaste and pleasantness) were obtained for the supplements and the ad libitum breakfast. A composite palatability score was calculated as the mean value of the palatability subscales [48].

### Blood sampling and biochemical analysis

In EXP1, participants rested in a semi-supine position for a minimum of five minutes before a cannula (Venflon, Becton Dickinson, Helsinborg, Sweden) was inserted into an antecubital vein by a trained phlebotomist. Blood samples were obtained at baseline (five min prior to each condition) and five, 30 and 60 min after supplement ingestion for the determination of plasma concentrations of acylated ghrelin, pancreatic polypeptide (PP), and peptide tyrosine tyrosine (PYY). Plasma C-reactive protein (CRP) concentration was also measured at baseline. At each time-point, samples were drawn into two pre-chilled 4.9 ml K3 ethylenediaminetetraacetic acid (EDTA) monovettes (Sarstedt, Germany). To prevent the degradation of acylated ghrelin, one monovette was prepared with a 50 μl solution of potassium phosphate buffer (PBS), P-hydroxymercuribenzoic acid (PHMB) and sodium hydroxide (NaOH). These monovettes were spun at 1000 x g for 10 min at 4 °C (ALC PK131R, Milan, Italy). The plasma supernatant was then pipetted into an Eppendorf tube and 100 μl of 1 M hydrochloric acid was added per millilitre of plasma to preserve acylated ghrelin (Hosoda et al., 2004). Following this, aliquots were spun at 1000 x g for 5 min at 4 °C and then stored in Eppendorf tubes at −80 °C for subsequent analysis. The accompanying monovettes were spun at 1000 x g for 10 min at 4 °C, with the resulting plasma supernatant pipetted into Eppendorf tubes for storage at −80 °C for subsequent analysis.

Commercially available enzyme-linked immunosorbent assay (ELISA) kits were used to determine plasma concentrations of acylated ghrelin (SPI BIO, Montigney le Bretonneux, France), PP (Millipore, Watford, UK), PYY (Millipore, Watford, UK), and CRP (IBL International GmbH, Germany), respectively. To eliminate interassay variation, samples from each participant were analysed in the same run. The within-batch coefficients of variation for each assay were 10.2%, 7.8%, 7.1% and 6.8% for acylated ghrelin, PP, PYY and CRP, respectively.

In a subset of participants (*n = 2*), untreated plasma samples were used to determine circulating amino acid concentrations. Prior to analysis, an internal standard (norleucine) was added to the plasma and proteins were removed by way of ultrafiltration. As previously described [7], plasma amino acids were analysed by gas chromatography mass spectrometry (GC-MS) after cation exchange and derivatisation as ethoxycarbonyl ethyl esters.

### Statistics

Data were analysed using SPSS for Windows (Version 22.0, IBM Corp., Armonk, NY). Time averaged area under the curve (AUC) values were calculated using the trapezoidal method. One-way repeated measures ANOVA was used to examine trial-based differences in energy intake and baseline CRP, as well as baseline and AUC values for appetite perceptions and plasma concentrations of acylated ghrelin, PP and PYY. Differences in the palatability of the supplements in experiment one was compared using paired t-tests and differences in the palatability of the breakfast meals in experiment two were compared using a one-way ANOVA. Normality was assessed by the Shapiro-Wilk test. Where significant effects were found, *post-hoc* analysis using Holm-Bonferroni correction for multiple comparisons was performed. The sample sizes employed within this study were deemed sufficient to detect a significant difference in energy intake between trials as the primary outcome measure for each experiment. Calculations were performed using G*Power with a meaningful difference in energy intake established as 500 kJ according to previous research [49], achieving 80% power with 10 participants, and based on the standard deviation for a similar ad libitum meal to that used within the present study [47]. Results in the text and tables are presented as mean (SD). Graphical representations of results are shown as mean (SEM) to avoid distortion of the graphs. Statistical significance in this study was accepted as *p ≤ 0.05*.

## Results

### Experiment one

#### Energy intake

**Fig. 2:**
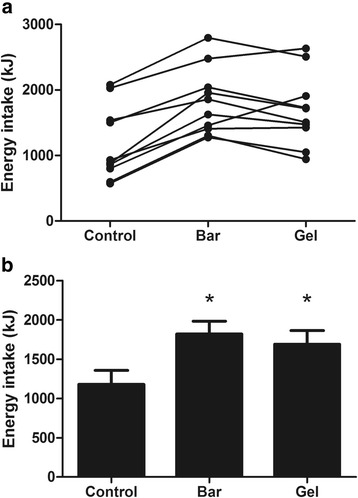
Total energy intake in the control, bar and gel trials (experiment one). Data are displayed as individual responses (**a**) and mean (SEM) (**b**), *n = 10*

Energy intake at the ALB was not significantly different between trials (CON 1179 ± 564, BAR 1254 ± 511, GEL 1206 ± 550 kJ; *P = 0.674*). However, total energy intake was significantly higher in the BAR and GEL than the CON after accounting for the energy content of the supplements (*P < 0.0005*; Fig. 2). Visual appeal, smell, taste, aftertaste and palatability of the breakfast did not differ between trials (all *P > 0.206*).

#### Appetite

**Fig. 3:**
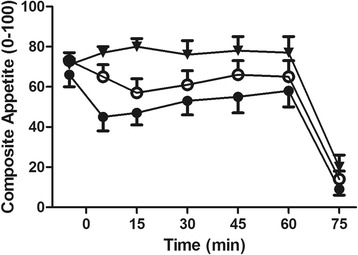
Composite appetite ratings in the control (▼), bar (●) and gel (○) trials (experiment one). Values are mean (SEM), *n = 10*

Composite appetite ratings did not differ between trials at baseline (CON 72 ± 10, BAR 67 ± 18, GEL 73 ± 13 mm; *P = 0.486*). One-way ANOVA revealed a significant time-averaged appetite AUC main effect (CON 72 ± 15, BAR 49 ± 19, GEL 59 ± 17 mm; *P = 0.008*; Fig. 3). Post-hoc analysis, demonstrated that appetite ratings were significantly higher in CON vs BAR (*P = 0.012)* and GEL vs BAR *(P = 0.009).* There was no difference between trials in AUC ratings for sweet (*P = 0.135*), savoury (*P = 0.930*), fatty (*P = 0.961*) or salty (*P = 0.448*) food preferences.

#### Plasma gut hormone concentrations

**Fig. 4:**
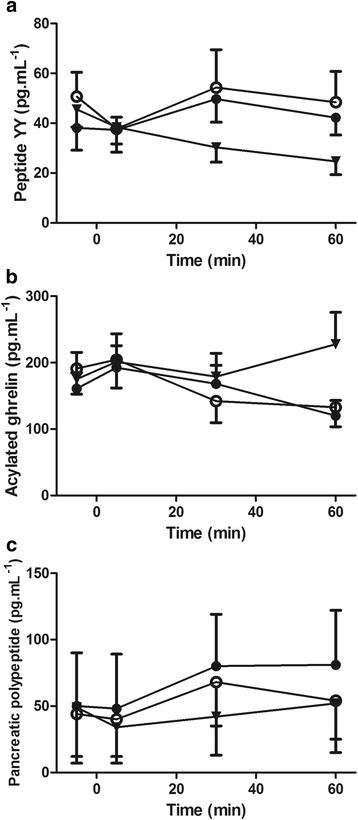
Plasma peptide YY (**a**), acylated ghrelin (**b**) and pancreatic polypeptide (**c**) concentrations in the control (▼), bar (●) and gel (○) trials (experiment one). Values are mean (SEM). *N = 10* for peptide YY and pancreatic polypeptide concentrations. *N = 9* for plasma acylated ghrelin concentrations

Plasma concentration of measured gut hormones are shown in Fig. 4abc. There were no significant differences between trials for fasting PYY (*P = 0.587*), acylated ghrelin (*P = 0.456*), PP (*P = 0.544*) or CRP concentrations (CON 1.14 ± 0.85, BAR 1.46 ± 1.35, GEL 1.27 ± 1.14 μg^.^mL^−1^; *P = 0.398*).

Time-averaged AUC for PYY did not differ between trials (CON 34 ± 19, BAR 46 ± 27, GEL 50 ± 31 pg^.^mL^−1^; *P = 0.236*; Fig. 4a). There was a tendency for lower time-averaged acylated ghrelin AUC in the BAR and GEL trials compared with CON (CON 204 ± 124, BAR 171 ± 83, GEL 169 ± 104 pg^.^mL^−1^; *P = 0.087*; Fig. 4b) and PP AUC was significantly higher in BAR and GEL than CON (CON 44 ± 102, BAR 72 ± 128, GEL 57 ± 102 pg^.^mL^−1^; *P = 0.020*; Fig. 4c).

#### Plasma amino acid concentrations

Mean plasma amino acid concentration during the three conditions is given in Table 2. Maximum plasma leucine concentrations were reached at 30 and 60 min post-ingestion in both the BAR (332.5 ± 65.4 and 409.1 ± 48.0 μMol^**.**^L^−1^ at 30 and 60 min respectively) and GEL (209.5 ± 77.5 and 390.2 ± 80.4 μMol^**.**^L^−1^ at 30 and 60 min respectively) conditions. The corresponding values for the CON were 64.7 ± 9.9 and 65.2 ± 9.4 μMol^**.**^L^−1^.

**Table 2:** Concentration of amino acids (μMol^**.**^L^−1^) in experiment one. Data are displayed as mean (SD), *n = 2.* Area under the curve (AUC)

Amino acids	Control (AUC)	Bar (AUC)	Gel (AUC)
Leucine	67.9 ± 6.6	276.5 ± 27.6	206.3 ± 7.9
Isoleucine	19.6 ± 2.4	55.6 ± 5.2	40.9 ± 0.0
Valine	115.7 ± 12.0	193.2 ± 32.1	153.7 ± 2.7
Alanine	274.0 ± 28.5	334.3 ± 54.7	308.7 ± 68.2
Glycine	92.1 ± 51.3	98.7 ± 44.6	90.3 ± 29.6
Proline	217.2 ± 26.5	243.7 ± 15.6	220.0 ± 7.6
Phenylalanine	20.8 ± 0.4	33.4 ± 1.5	27.5 ± 0.8

#### Palatability of supplements

Paired t-tests revealed higher ratings of visual appeal (*P < 0.0005*) and smell (*P = 0.035*) for the BAR compared with the GEL (visual appeal: BAR 80 ± 17 versus GEL 23 ± 20; smell: BAR 68 ± 16 versus GEL 45 ± 21). There were no differences between supplements for ratings of taste, aftertaste or overall palatability (all *P > 0.277*).

### Experiment two

Composite appetite ratings did not differ between trials at baseline (CON 77 ± 11, BAR 70 ± 15, GEL 70 ± 18 mm; *P = 0.141*).

**Fig. 5:**
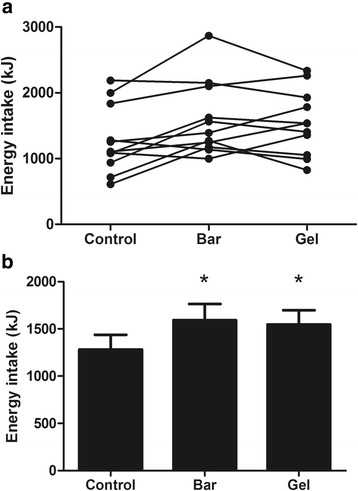
Total energy intake in the control, bar and gel trials (experiment two). Data are displayed as individual responses (**a**) and mean (SEM) (**b**), *n = 11*

Energy intake at the ALB was significantly higher in the CON compared with the BAR and GEL (CON 1282 ± 513, BAR 1026 ± 565, GEL 1064 ± 495 kJ; *P = 0.028*). However, total energy intake was significantly higher in the BAR and GEL than the CON after accounting for the energy content of the supplements (*P = 0.007*; Fig. 5).

Visual appeal, smell, taste, aftertaste and palatability of the breakfast did not differ between trials (all *P > 0.159*).

### Experiment one and two

Participants in both experiments complied fully with the dietary and exercise standardisation requirements for the 24-h period preceding each testing trial. In cases where participants did not replicate the 24-h diet and exercise records completed for the first experimental trial, testing was rearranged.

## Discussion

To our knowledge, this is the first study to demonstrate that supplementation of two unique essential amino acid blends, administered as either a bar or a gel, enabled older women to increase energy intake regardless of whether the supplements were taken one hour before or alongside an ad libitum breakfast meal (ALB). The composition of the supplements was optimised for the target population with each supplement containing 7.5 g of EAAs, the equivalent of approximately 15 g of high quality protein, and high leucine content (~3 g). The current composition did not negatively affect palatability, smell, taste and aftertaste when consumed alongside a typical porridge-based breakfast meal. Therefore, our data suggests that the current formulations and modes of ingestion can be an effective alternative dietary means of protein ingestion, for women who need to enhance total daily protein intake or a high quality protein intake per meal, without negatively affecting energy intake.

In experiment one, energy intake at ALB was not significantly different between conditions while the additional energy provided by either the bar or the gel (565 and 477 kJ respectively) contributed to achieving significant elevations in total energy intake. Supplementation one hour before the ALB did not result in any subsequent reductions in food volume, as has shown in other cases where use of dense nutritional supplements results in compensatory caloric redistribution [50]. In our study, a small to moderate reduction in volume of food eaten was expected, when the supplements were consumed one hour before the ALB, yet this did not happen. As expected, appetite ratings during the one-hour post-supplement ingestion were significantly higher in the control condition. However, the magnitude of increase was not adequate to cause subsequent reductions in food consumed during the ALB. Neither the bar nor the gel affected the food cravings of participants, since no differences were observed between the preferences for sweet, savoury, fatty, or salty foods.

Food protein sources or protein supplements may increase satiety and reduce energy intake [17, 31, 32]. Therefore, key objective was to develop nutritional supplements with minimal satiating effects and optimised EAAs concentration. The tested supplements were enriched with EAAs instead of protein in an attempt to address deficits in protein intake without reducing energy intake. This is important since protein-energy malnutrition is high in institutionalised older individuals [51–53], where large dietary deficiencies [38] puts them at greater risk for complications and death in proportion to the degree of protein-energy deficiencies [54]. It is also true of community living older populations where the prevalence of malnutrition has been reported as 5–8% [55] and up to 66% considered at nutritional risk [56].

The satiating effects of dietary protein, which results in subsequent reductions in energy intake, partly through increases in peptide tyrosine-tyrosine (PYY), and post-prandial fullness [17, 32] pose additional challenges. In contrast, our own study indicated no significant differences in PYY concentration between conditions. Alternatively, plasma acylated ghrelin concentrations tended to be lower after the consumption of both the bar and the gel, and PP concentrations were significantly elevated during these conditions. Such changes would be expected as means to aid digestion [57] but were much lower than what would typically be observed following consumption of a high protein meal [58]. The lower appetite perceptions observed in concordance with these changes in appetite-regulating hormones was not sufficient to alter energy intake during the ALB, which suggests that the supplements provided in this study may help to increase overall energy intakes.

Our data showed that large increases in plasma concentration of amino acids reached peak values within 30–60 min post-ingestion. This highlights the highly efficient digestion and absorption rates of both supplements comparable to supplementation with amino acids [59] or higher amounts of high quality protein [44, 60]. One of the reasons whey protein elevates muscle protein synthesis to a greater extent than lower quality proteins [44, 61] is due to higher leucine content [62, 63], which plays a crucial role in the regulation of muscle protein synthesis [39]. In our case, leucine concentration values were much higher than those of high quality proteins therefore the current supplements could be an effective means to optimise muscle protein synthesis rates in older people. Indeed, studies have shown that muscle protein synthetic rate in the old is higher with increasing leucine content either in mixtures of EAAs [41] or whey protein [43, 44]. Furthermore, studies have provided good evidence for the prophylactic role of EAAs [33, 64] or EAAs enriched with leucine [7, 65]. More importantly, it has been observed that older individuals with higher leucine intakes maintain lean mass over a six year period while those with lower intakes do not [66].

In experiment two, energy intake at ALB was significantly lower than the control, which demonstrated adequate sensitivity of the breakfast meal to detect changes in energy intake. Nevertheless, total energy intake was significantly higher in the experimental conditions when energy from the supplements was accounted for. Therefore, both the bar and the gel can be taken alongside food as means to enhance total energy intake and the anabolic effect of food. Ingestion of the supplements alongside food can be particularly effective since older individuals tend to receive at breakfast and lunch less than 23 g of protein per meal [67] with protein deficiencies being much larger at breakfast. Recommendations suggest the amount of protein per meal should be at least 25–30 g [17, 18] or approximately 0.4 g^.^kg^-1.^BM^−1^ [68], with high leucine content [69]. Further studies have provided additional support for the need for higher protein intake per meal where it has been shown that one to two daily meals containing 30–45 g of protein each were associated with more favourable gains in lean tissue mass and strength of older men and women [14]. Similarly, Gregorio et al. [19] also observed that older women with higher dietary protein intakes had better physical performance and body composition than women with lower protein intakes. Supplementation with whey protein (30 g daily for a period of 2 years) in healthy older women [37] on the other hand did not result in gains in muscle mass and physical function and this may be because protein intakes at baseline were higher than current recommendations. However, it is not known if there was a reduction in total energy intake in the protein group due to the potential satiating effects of protein [17, 32]. The current supplements can facilitate an overall increase in protein and leucine intake without negatively affecting energy intake. In line with experiment one the supplements were received well by all participants. The palatability of the breakfast was considered “good” on scoring, and not affected by concurrent consumption of the supplements during the meal. Despite an adequate sample size for the current study, there is a need for further research to confirm the generalisability and reproducibility of our findings in larger clinical and non-clinical populations, as well as in men. Using a validated breakfast meal, our findings confirm that both supplements can facilitate an acute increase in protein and energy intake. However, it is still unknown how daily intakes may be affected when the supplements are taken alongside other main meals of varied composition.

## Conclusions

In conclusion, our results demonstrate that ingestion of oral nutritional supplements containing 7.5 g of EAAs enriched with 40% leucine (3 g) increased total energy intake whether consumed one hour before or during an ad libitum *breakfast*. This may represent an effective nutritional means for addressing protein and total energy deficiencies in older women.

## Abbreviations

ALB: Ad libitum breakfast
AUC: Area under the curve
BAR: Bar
CON: Control
DBP: Diastolic blood pressure
EI: Energy Intake
ELISA: Enzyme-linked immunosorbent assay
ES: Effect Size
Essential Amino Acids: EAAs
EXP: Experiment
GEL: Gel
IPAQ: International Physical Activity Questionnaire
PI: Protein intake
RHR: Resting heart rate
SBP: Systolic blood pressure
